# Multidisciplinary follow-up in a patient with Morgagni hernia leads to diagnosis of Marfan syndrome

**DOI:** 10.1186/s13052-024-01643-8

**Published:** 2024-05-07

**Authors:** Ester Capecchi, Roberta Villa, Alessandro Pini, Maria Iascone, Laura Messina, Paola Francesca Ajmone, Fabio Mosca, Silvana Gangi, Maria Francesca Bedeschi

**Affiliations:** 1 Neonatal Intensive Care Unit, Fondazione IRCCS Ca’ Granda Ospedale Maggiore Policlinico, Via Francesco Sforza, 28, 20122 Milan, Italy; 2 Medical Genetic Unit, Fondazione IRCCS Ca’ Granda Ospedale Maggiore Policlinico, Milan, Italy; 3 Cardiovascular Genetic Unit, IRCCS Policlinico San Donato, San Donato Milanese, Milan, Italy; 4 Laboratory of Medical Genetics, ASST Papa Giovanni XXIII, Bergamo, Italy; 5 Pediatric Physical Medicine & Rehabilitation Unit, Fondazione IRCCS Ca’ Granda Ospedale Maggiore Policlinico, Milan, Italy; 6 Child and Adolescent Neuropsychiatric Service (UONPIA), Fondazione IRCCS Ca’ Granda Ospedale Maggiore Policlinico, Milan, Italy; 7 Department of Clinical Sciences and Community Health, University of Milan, Milan, Italy

**Keywords:** Congenital diaphragmatic hernia, FBN1 mutation, Marfan syndrome

## Abstract

**Background:**

congenital diaphragmatic hernia (CDH) is a birth defect occurring in isolated or syndromic (chromosomal or monogenic) conditions. The diaphragmatic defect can be the most common one: left-sided posterolateral, named Bochdalek hernia; or it can be an anterior-retrosternal defect, named Morgagni hernia. Marfan syndrome (MFS) is a rare autosomal dominant inherited condition that affects connective tissue, caused by mutations in fibrillin-1 gene on chromosome 15. To date various types of diaphragmatic defects (about 30 types) have been reported in association with MFS, but they are heterogeneous, including CDH and paraesophageal hernia.

**Case presentation:**

We describe the case of a child incidentally diagnosed with Morgagni hernia through a chest X-ray performed due to recurrent respiratory tract infections. Since the diagnosis of CDH, the patient underwent a clinical multidisciplinary follow-up leading to the diagnosis of MFS in accordance with revised Ghent Criteria: the child had typical clinical features and a novel heterozygous de novo single-base deletion in exon 26 of the *FBN1* gene, identified by Whole-Exome Sequencing. MFS diagnosis permitted to look for cardiovascular complications and treat them, though asymptomatic, in order to prevent major cardiovascular life-threatening events.

**Conclusion:**

Our case shows the importance of a long-term and multidisciplinary follow-up in all children with diagnosis of CDH.

## Background

Congenital diaphragmatic hernia (CDH) is a relatively common major birth defect with estimated incidence between 1/2.000 and 1/3.000 live- and still-births [1–3]. Left-sided posterolateral diaphragmatic defects, named Bochdalek hernias (BH) are the most common and occur in 85% of all cases, followed by anterior-retrosternal defects, named Morgagni hernias (MH) [4]. BH is usually diagnosed in the prenatal period on ultrasound or soon after birth in a newborn with severe respiratory distress due to pulmonary hypoplasia [3, 4]. MH occurs more often on the right hemidiaphragm and is usually incidentally diagnosed in adulthood because of its asymptomatic course. When symptoms occur they include respiratory complaints or gastrointestinal symptoms [4, 5].

Approximately 40 to 50% of cases of CDH occur in combination with additional congenital abnormalities (complex CDH), such as congenital heart diseases, either as a part of a syndrome or a non-syndromic association [1, 3]. The frequency of syndromic forms of CDH ranges from 14 to 44% [1] including chromosomal defects and monogenic conditions [6].

Marfan syndrome (OMIM #154,700, MFS) is among the monogenic conditions with CDH as described features.

MFS is a rare (reported incidence of one in 5.000–10.000 births) autosomal dominant inherited condition that affects connective tissue, caused by mutations in fibrillin-1 (*FBN1*, OMIM*13,479) gene on chromosome 15. Fibrillin-1 is the main protein component of extracellular microfibrils that are thought to contribute to the formation and maintenance of elastic fibres.

Cardinal features of MFS involve the skeletal, cardiovascular and ocular systems; the signs and symptoms of MFS can vary greatly, even among members of the same family [7–10]. Clinical diagnosis is based on the 2010 revised Ghent criteria, which replaces the previous 1996 nosology arising from the 1988 revision of the Berlin diagnostic criteria [7]..

Various types of CDH have been reported in association with MFS, especially in the early onset MFS or neonatal form (nMFS) [11]. MH appear to be a rarer entity associated to MFS reported to date in only 5 cases (2 children and 3 adults) [11–15]. Herein, we describe an additional case of a 5-years-old-boy diagnosed with MH carrying a novel *de novo* pathogenic variant in *FBN1*.

.

## Case presentation

Our patient was an Italian male born at term by spontaneous delivery, after uncomplicated pregnancy. The baby was the first child of non-consanguineous parents. The mother was 32 years old and was on L-thyroxine replacement therapy for hypothyroidism due to Hashimoto’s thyroiditis. The father (35years old) was in good health and had another healthy son. The family history was remarkable for a paternal uncle, dead at birth because of bilateral renal agenesis, and a paternal aunt born deaf because of cochlear agenesis. At birth, the weight was 3.530 g (64th percentile), the length was 50 cm (52nd percentile) and the head circumference was 36 cm (92nd percentile). Apgar score was 10 at 1st and 5th minute. The child had physiologic perinatal period and normal psychomotor development.

During the first year of life, he presented recurrent respiratory tract infections.

At 16 months of age, due to slowly-resolving bronchopneumonia diagnosed 15 days earlier, he presented to the Emergency Department where he performed a chest X-ray revealing the presence of intestinal loops in the mediastinum, suggesting a diaphragmatic hernia. The child was then referred to our Surgical Department where, a computed tomography scan of the chest confirmed the anterior herniation of the diaphragm (MH). The cardiologic evaluation and the abdominal echography were normal. A genetic evaluation showed minor facial anomalies such as downlanting palpebral fissure, epichantal folds, protruding ears, malar hypoplasia, full lip, retrognathia, ogival palate, pectus carinatum, clinodactyly of the 5th finger, slight hypermobility of distal interphalangeal joints, normal development milestones and anthropometric parameters with height at 75-90th percentile. A specific pattern of syndrome was not recognized. An array-CGH (Array-Comparative Genomic Hybridization) (8 × 60 K Kit) was unremarkable. The patient underwent surgical repair through laparoscopic transabdominal route which was followed by substantially uneventful recovery.

After the discharged, a multidisciplinary follow-up was started to provide the necessary care for the possible CDH-complications.

Over the next months, he was diagnosed with bilateral flat and valgus feet, corrected with orthopedic insoles, and vitamin D deficiency for which supplementation was started. The surgical follow-up was normal; the respiratory function tests resulted concordant with values of children with CDH and the first ophthalmologist visit documented ocular divergence in the upward gaze.

**Fig. 1:**
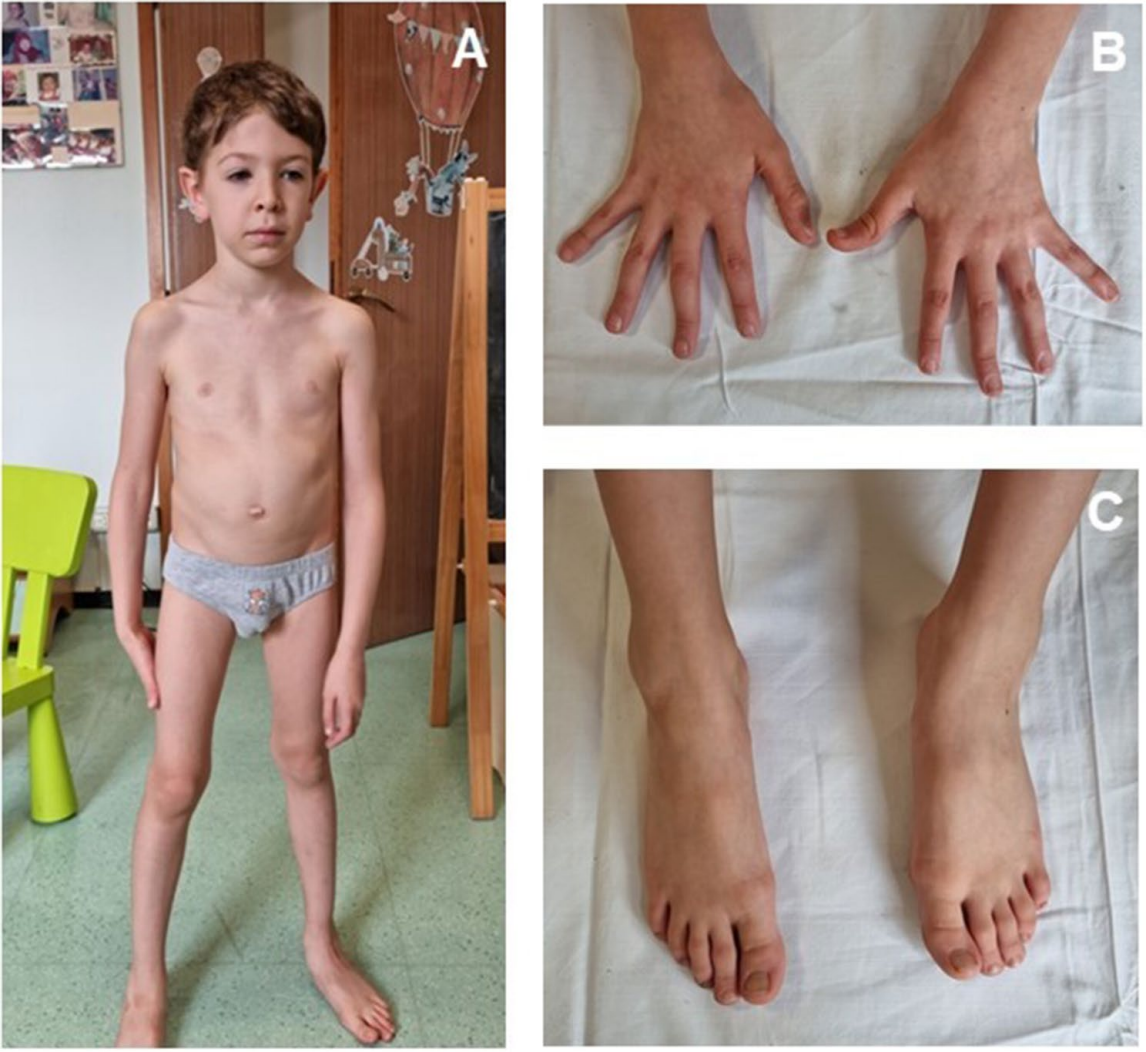
Evolution of the patient at 5 years of age: **A**: shows marfanoid habitus, peculiar facial anomalies, and pectus carinatum. **B** and** C** indicate the aracnodactyly of the hands and feet respectively

At 32 months of age, the proband and his parents underwent to trio-based whole-exome sequencing (WES). WES identified the frameshift variant NM_000138.4:c.3089del; (p.Asn1030Metfs*5) in exon 26 of *FBN1*, which was further confirmed by Sanger sequencing. The variant occurred *de novo* in the proband. It was absent in population databases (i.e. gnomAD) and is predicted as disease causing by several bioinformatics tools, consistent with the early termination of protein translation. According to the ACMG/AMP guidelines, NM_000138.4:c.3089del would be classified as pathogenic based on the criteria involving PVS1, PM2, PM6 and PP5. At 38 months of age, the transthoracic echocardiography showed ectasia of the aortic root to the sinuses of Valsalva (Z-score 2,35) with normal aortic valve, mitral valve prolapse (MVP) with mild regurgitation and initially thickened flaps. An angiotensin converting enzyme (ACE) inhibitor was prescribed to the child in order to regularize the growth rate of the aortic root and reduce the ratio between aortic root and body surface over time. Moreover, the ophthalmologic examination was normal. In accordance with revised Ghent Criteria MFS diagnosis was established in the patient. The last genetic evaluation at 5 year-old confirmed minor facial and skeletal anomalies  , marfanoid habitus, slight hypermobility of distal interphalangeal joints and normal psychomotor development (Figure 1).

## Discussion and conclusions

MFS is a multiorgan disorder affecting eyes, skeleton, heart and arteries, dura and lungs; it is notable for its phenotypic variability in age at onset, organ involved and severity of clinical manifestation, both among and within affected families.

MFS is diagnosed according to the revised Ghent criteria. In 2010 the Ghent Criteria were updated adding genetic testing of *FBN1* and the presence of a causative *FBN1* variant as key criteria in the diagnosis of MFS besides major clinical features as aortic root dilatation and ectopia lentis [10].

Though the use of revised Ghent Criteria improves the diagnostic yield of MFS, in young children the diagnosing MFS is still very challenging as some of its clinical manifestations are age dependent.

Our patient satisfies the criteria showing aortic root dilatation and causal mutation of *FBN1* gene. He also presents a Systemic Score of 3: plain flat foot (1 points); peculiar facial anomalies with dolichocephaly, down-slanting palpebral fissures, malar hypoplasia and retrognathia (1 point) and MVP (1 point). Moreover, arachnodactyly is evident in both hands and feet.

Aortic root dilatation, initially occurring at the sinuses of Valsalva, is the main cardiovascular manifestation of MFS, which can lead to life-threatening sequelae (i.e. aortic dissection).

The reported prevalence in young children with MFS varies among studies from 50 to 83% and increase with time. Aburawi et al. estimated an aortic root dilatation prevalence of 35% at 5 years [16]. In our patient, aortic dilatation was described at 3 years of age, while the previous echocardiography at 16 months of life was normal.

In our report, WES analysis revealed a *de novo* heterozygous single-base deletion in in exon 26 of *FBN1* gene ( NM_000138.4:c.3089del) producing an altered reading frame and generating a premature termination of translation. The mutation appears to be novel; nevertheless, according to the current ACMG/AMP guideline it is possible to definite it causative.

To date, few genotype-phenotype correlations for *FBN1* mutations have been uncovered. In a series of 570 adult patient with MFS, those with FBN1 haploinsufficiency mutations had a significantly increased risk for any aortic complications (such as aortic dissection, aortic surgery) than those with missense (dominant-negative) mutations (adjusted hazard ratio 1.6; 95%CI 1.1–2.7) [17]. In addition, Baudhuin L. M. et al. found that the ratio of *FBN1* truncation mutations was also higher in the aortic events in patients affected by MFS [18]. It is also reported in some previous studies that patients harbouring truncating mutation show lower likelihood of diagnosis of ectopia lentis versus patients with missense mutations [19]. This data supports the absence of any actual ocular manifestations in our patient.

Variants in exons 24–32 are typically associated with a severe form of MFS rapidly progressive [], previously named nMFS [9]. However, some patients with severe MFS lack mutations in this region and many patients with mutations in this region have classic or mild variants of MFS. Likewise, we identified in our proband a *FBN1* pathogenic variant in exon 26 which seems to have a little prognostic value as our patient presents a mild phenotype of MFS.

Although gastrointestinal tract manifestations are not listed in Ghent nosology as a diagnostic sign of MFS, many MFS patients’ present gastrointestinal tract involvement such as liver cysts, biliary duct ectasia, CDH, intussusception, volvulus, as well as functional disorders.

To the best of our knowledge 24 cases of CDH associated with MFS have been reported so far and listed in Table 1. The definitive pathogenetic explanation for the association of CDH and MFS is still limited. However, it is thought that diaphragmatic defect is caused by a deficiency of fibrillin-1 during fetal development of diaphragm that may disrupt mesenchymal cell proliferation, causing malformed muscle tissue as well as abnormally lax oesophageal hiatus and lax gastric ligamentous attachments resulting in diaphragmatic defects, as was documented in the foetuses of rats nitrofen-induced CDH model [20].

**Table 1:** Defect of the diaphragm and FBN1 mutation

	Article	FBN1 Mutation	Family history	Age at CDH diagnosis	Type of CDH
1	Parida et al., 1997 [26]	n.a.	positive	birth	PHE
2	Jacobs et al., 2002 [27]	c.3143T > C	negative	birth	Right-BH
3	Yetman et al., 2003 [13]	n.a.	n.a.	22 years	Right-MH
4	Peterson et al., 2003; c*ase 1* [28]	n.a.	positive	20 days	PHE
5	Peterson et al., 2003; c*ase 2* [28]	n.a.	positive	birth	PHE
6	Peterson et al., 2003; *case 2 mother* [28]	n.a.	n.a.	3 months	PHE
7	Al-Assiri et al., 2005 [29]	n.a.	positive	prenatal	PHE
8	Barakat et al., 2005 [30]	n.a.	positive	43 years	Right hemidiaphragm agenesia
9	Keswani et al., 2007 [31]	n.a.	positive	35 years	PHE
10	Jetley et al., 2009; *case 3* [32]	n.a.	positive	4 months	PHE
11	Jetley et al., 2009; c*ase 8* [32]	n.a.	positive	birth	PHE
12	Martinez-Lesquerux et al., 2010 [14]	n.a.	n.a.	53 years	Left-MH
13	Ayse Esin et al., 2010 [4]	n.a.	positive	9 years	Right-MH
14	Herman et al., 2013 [33]	n.a.	positive	birth	PHE
15	Thakur et al., 2017 [34]	n.a.	negative	25 years	PHE
16	Beck et al., 2015; f*ather* [2]	c.4969_4970insA	n.a.	10 years	PHE
17	Beck et al., 2015; s*on* [2]	c.4969_4970insA	positive	2 months	Right-BH
18	Laumonerie et al., 2015 [12]	c.733 C > T	positive	2 months	MH
19	Stępiński et al., 2016 [35]	n.a.	n.a.	26 years	PHE
20	Kothari et al., 2017 [36]	n.a.	n.a.	35 years	PHE
21	S. Somasundaram et al., 2019 [15]	n.a.	n.a.	45 years	MH
22	Gupta et al., 2020; *sibling 1* [37]	n.a.	positive	< 3 years	PHE
23	Gupta et al., 2020; *sibling 2* [37]	n.a.	positive	< 3 years	PHE
24	Hou et al., 2021 [38]	n.a.	n.a.	77 years	PHE
25	Present case	c.3089del	Negative	16 months	MH

*FBN1*: fibrillin-1 gene; n.a.: not available; CDH: congenital diaphragmatic hernia; PHE: paraesophageal hernia; BH: Bochdalek hernia; MH: Morgagni hernia

As showed in Table 1, PHE is the most common type of diaphragmatic defects reported in patients with MFS. The CDH doesn’t seem to be associated with a specific MFS phenotype. In our case the patient had a MH, of which only 5 cases in association with MFS were previously described. The age of MH diagnoses ranging from 2 month to 53 years with a median age of 22 years as the majority of patients tend to be pauci- or a-symptomatic.

Yetman et al. reported a MH in a 22-year-old man with MFS presented with acute dyspnea secondary to bowel herniation, who, similarly to our case, was initially incorrectly diagnosticated as an acute pneumonia. Later, the diagnosis of MFS in this patient was stated as the presence of aortic root dilatation, mitral valve prolapse, ocular lens subluxation and skeletal anomalies [13]. Similarly, Ayse Esin et al. described the diagnosis of MH in a 9-year-old female presenting with respiratory symptoms. a positive family history of MFS and, at physical examination, MFS facial appearance, tall stature and a systolic murmur that prompts the diagnosis of aortic root dilatation and mitral and tricuspid valve prolapse[4]. Laumonerie et al. described a 3 month-old female with phenotype and family history strongly suggestive for MFS presenting with MH [12]. This case demonstrates an affinity with our report as MH was the first feature of MFS and the infant showed cardiac involvement requiring for targeted therapy already at a young age.

According to cardiological involvement showed in some cases, we cannot say that patients with MFS and MH have less severe clinical phenotype; rather we highlight how an early diagnosis of MFS is essential to prevent cardiovascular complications, in order to increase life expectancy of patients [21].

MFS prognosis is related to aortic root dissection, a complication related to aortic root dilatation. A French multicentre study assessed the incidence of cardiovascular events and their associated risk markers in children with MFS and set that a close follow up and timely prophylactic surgery during childhood prevent dissection and that aortic root Z score and aortic valve regurgitation seem interesting risk markers for cardiovascular events and progressive aortic root dilation [22].

In our case, the child has aortic root dilatation and mitral valve prolapse at the age of 4 years and a prophylactic treatment was started.

Traditionally B-blockers and angiotensin receptor blockers (ARBs) are prescribed at the time of diagnosis with MFS at any age or upon appreciation of progressive aortic dilatation. These drugs are expected to reduce the risk of aortic dissection reducing the systolic ejection impulse and consequently hemodynamic stress on the aortic wall [7]. Chiu et al. demonstrated that the combination of B-blockers and losartan affords better protection against aortic root enlargement than B-blockers alone in children with MFS [23]. In the same yeara study conducted in a small cohort of young patients with MFS proved a significant improvement with losartan monotherapy in all affected proximal aortic segments, with a better response to therapy when started at an earlier age and with a longer therapy duration [24].

As alternative to B-blockers and ARBs, calcium antagonists and ACE inhibitors have been considered. Enalapril has been seen to improve aortic distensibility and reduce the rate of aortic dilatation compared with B-blockers in a small not randomized clinical trial in children and adolescents [25]. According to these findings Enalapril therapy was started in our case.

Our experience teaches the importance of a multidisciplinary follow up, particularly genetic and pediatric one, for a child with an occasional anterior CDH and some minor dysmorphisms. The continuous clinical evaluations of the patient allowed to make diagnosis and to set up an early cardiological therapy in an asymptomatic child that we hope will prevent the even fatal complications of MFS.

## Data Availability

Not applicable to this article as no datasets were generated or analysed during the study.

## Abbreviations

CDH: congenital diaphragmatic hernia
BH: Bockdalek hernia
MH: Morgagni hernia
MFS: Marfan syndrome
FBN1: fibrillin-1
nMFS: neonatal Marfan syndrome
PHE: paraesophageal hernia
Array-CGH: Array-Comparative Genomic Hybridization
CNVs: Copy Number Variants
WES: whole-exome sequencing
MVP: mitral valve prolapse
ACE: angiotensin converting enzyme
ARBs: angiotensin receptor blockers
